# Functional Optical Nano/Micromaterials

**DOI:** 10.3390/ijms24087458

**Published:** 2023-04-18

**Authors:** Won-Yeop Rho, Bong-Hyun Jun

**Affiliations:** 1 School of International Engineering and Science, Jeonbuk National University, Jeonju 54896, Republic of Korea; rho7272@jbnu.ac.kr; 2 Department of Bioscience and Biotechnology, Konkuk University, Seoul 05029, Republic of Korea

The interaction between light and optical materials is central to science, as these materials possess remarkable physical, chemical, and photonical characteristics. Currently, research on a wide range of applications has been conducted based on various functional optical materials [1,2,3,4]. This Special Issue will explore a range of original contributions detailing a range of functional nano/micromaterials with unique properties. These include metal nanomaterials, quantum dots and carbon materials, among others. It also covers various functional materials and their respective applications in optics. In this Special Issue, we present six papers that discuss development related to nano/micromaterials-based approaches for optical functionality enhancement purposes.

Metal porphyrinates, including copper(II) etioporphyrinate, have characteristic optical properties, and thus have been applied to optical or electron-optical devices [5,6,7]. Chernyadyev et al. studied the luminescent transition properties of copper(II) etioporphyrinate (Cu-EtioP), which presented a new possibility for a luminescence temperature sensor as part of an active OLED layer [8]. In their study, Cu-EtioP showed thermal sensitivity of phosphorescence spectra and demonstrated that Cu-EtioP was a promising material for fluorescent thermosensors such as palladium(IV) and rhodium(III).

Materials using nanopatterns also improve the transmittance of light energy [9,10,11]. Yang et al. tried to improve the energy conversion efficiency (ECE) of perovskite solar cells by introducing mesoporous TiO_2_ (mp-TiO_2_) nanopatterns with an imprinting method [12]. The nanopatterned mp-TiO_2_ led to an effective ECE enhancement of perovskite solar cells by enhancing the transmittance of solar light at wavelengths above 400 nm that affect electron generation. They also suggested that a nanopatterning method involving simple imprinting can introduce nanopatterns optimally for other types of solar cells, enabling development for better solar cell performance.

When gold nanoparticles were embedded and grown on silica nanoparticles with nanogaps, various colors (pink to dark blue) were represented due to the strong plasmonic coupling [13]. Seong et al. reported the synthesis of gold nanoparticles embedded silica nanoparticles (SiO_2_@Au NPs), controlling the size of gold nanoparticles and their application [14]. They aimed to control the size of gold nanoparticles by adjusting the amount of added gold precursor after attachment of around 2.5 nm-sized gold nanoparticles in seed form, allowing fine control of the size of gold nanoparticles. Fabricated SiO_2_@Au NPs showed unique colors according to the size of embedded gold nanoparticles. Furthermore, since gold nanoparticles have unique peroxidase-like activity, SiO_2_@Au NPs could acte as easy-to-handle nanozymes, which were reusable and highly stable under long-term storage. Based on these characteristics, hydrogen peroxide could be detected sensitively with SiO_2_@Au NPs.

To enhance the fluorescence signal of quantum dots (QDs), novel nanoparticles consisting of a silica core, multiple numbers of embedded QDs and a silica shell were developed [15,16]. As part of this development, Hahm et al. embedded QDs onto the surface of silica shell-coated Ag nanoparticle-assembled silica nanoparticles (SiO_2_@Ag@SiO_2_@QD) [17]. The study using SiO_2_@Ag nanoparticles was able to demonstrate a method of enhancing fluorescence through the simple introduction of a 10–14 nm thin SiO_2_ layer into the SiO_2_@Ag core–shell nanosphere. Furthermore, it enabled the optimization and improvement of SiO_2_@Ag-based optical sensors with surface-enhanced Raman scattering (SERS) and metal-enhanced fluorescence (MEF) effects. Kim et al. reported an improved single-particle tracking (SPT) method using silica-coated quantum dot-embedded silica nanoparticles (SiO_2_@QDs) as an alternative to traditional organic fluorophores or single quantum dots. SiO_2_@QDs has improved stability, biocompatibility, and brightness, enabling long-term intracellular tracking without significant photobleaching. SiO_2_@QDs labeling efficiency was retained for 96 h with minimal cytotoxicity and did not impair cell function. This technique was successfully used to visualize in situ endothelial vessel formation without real-time staining [18].

The two reviews using nanoparticles presented in this Special Issue provide insight into how to advance previous studies and suggest potential applications of nano/micromaterials-based sensors in our everyday life. A review by Tim and Blaszkiewicz et al. introduces an optical sensor based on surface-enhanced Raman scattering using assemblies of metal nanoparticles, resulting in hotspot formation and energy resonance [19]. The ability to generate local surface resonance plasmon bands and plasmonic hot spots of metal nanoparticles can be influenced through chemical reaction control, which is related to the optical properties of the sensor. In this review, the study of controlled various shapes and patterned metal particles suggests that the SERS nanoplatform will soon present a breakthrough that can be applied to everyday life.

Methods of utilizing nano QDs for optical sensor applications in the biomedical field are also reviewed [20]. Since QDs use hydrophobic ligands, hydrophilic modification must be applied to living organisms, and Pham et al. introduce a method to overcome the disadvantages of QDs using silica coating. In this review, silica coating allows QDs to be easily applied to fields such as agriculture, environmental sciences, biology, biosensing, in vitro analysis and imaging, etc. This indicates that applications of this technology are possible.

Functional optical nano/micro materials are becoming increasingly important in a variety of industries, from medical diagnostics to consumer products. They can be introduced into various surfaces through particle synthesis and patterning to contribute to the enhancement of the resulting product. This Special Issue focuses on six technologies for engineering functional optical nano/micromaterials (https://www.mdpi.com/journal/ijms/special_issues/funtional_optical_materials) which are essential for advanced materials such as in vivo nanodiagnostics [21].

The papers discussed within this issue provide an overview of how these technologies will be applied, ranging from sensing applications that make our lives more convenient, precise control over material properties and even greater accuracy when used with other existing techniques such as spectroscopy or microscopy imaging techniques. The use of functional optical nano/micromaterials enables us to apply these technologies across multiple disciplines while still achieving high levels of precision and accuracy due to their small size yet powerful capabilities.
